# Ramadan fasting in hemodialysis population: single-center study

**DOI:** 10.1186/s43162-022-00150-8

**Published:** 2022-08-17

**Authors:** Ahmed Abdelmoniem Emara, Ahmed Hamed Ghareeb, Mahmoud Fayez, Reem Mohsen Elsharabasy

**Affiliations:** Department of Nephrology, Ain Shams University, Cairo, Egypt

**Keywords:** Hemodialysis, Fasting, Ramadan, Kidney disease

## Abstract

**Background:**

Fasting Ramadan is one of the fundamental pillars of Islam. Although sick people are excluded from this duty, some of the hemodialysis patients insist to fast to enjoy the spiritual nature of the holy month.

**Objectives:**

To monitor the tolerability of fasting Ramadan among the hemodialysis population.

**Methodology:**

One hundred ninety-nine prevalent hemodialysis (HD) patients participated in the study and were allocated to 3 groups according to their fasting decision (complete, partial, and non- fasting). Basic demographic and laboratory data were collected before the start of the holy month; monitoring any inter or intradialytic complications or events during the holy month was done in addition to dry weight monitoring before and at the end of the month.

**Results:**

One hundred ninety-nine HD patients were included (97 males, mean age 45 ± 15 SD). Patients were divided based on their fasting state into 3 groups: compete fasting 28 (14%), partial fasting 88 (44%), and non-fasting 83 (42%). Out of 116 total fasting patients, only 4 patients (3.4%) developed complications (intradialytic hypotension (IDH) and muscle cramps) during dialysis. On the other hand, 3 patients experienced improvement of IDH; also, one patient reported improvement in dyspepsia. We noted a significant reduction in dry weight in the complete and partial fasting groups (*P* < 0.001 for both), unlike the non-fasting group (*P* = 0.75).

**Conclusion:**

We may conclude that fasting Ramadan in hemodialysis patients whether complete or partial fasting may be tolerated by most of patients and was associated with a significant reduction in dry weight.

## Introduction

Ramadan is a sacrosanct month for all Muslims around the world; it is the ninth lunar month of Islamic calendar. Fasting Ramadan is the fourth of the five pillars of Islam, a religious duty on every adult, sane, healthy Muslim, who are required to fast from dawn to sunset. Severely ill and those with significant health problems, menstruating females, pre-pubertal children, and travelers are exempted from fasting [1]; thus, hemodialysis (HD) patients are given a chance not to fast due to their underlying health condition; however, due to the exquisite spiritual nature of the holy month, some insist on fasting [2].

The effects of Ramadan fasting on physiological and biomedical markers among healthy individuals have been widely studied; this is usually feared due to the major shift in the timings and components of the patients’ meals during breaking their fast, such as the abundancy of Ramadan’s festive food, dried fruits, juices, and more importantly the fluid intake, which raises healthcare professionals concern about the safety of Ramadan fasting in chronic kidney disease (CKD) and end-stage kidney disease (ESKD) patients under their care. However, research evidence suggests that Ramadan fasting is tolerable and safe for healthy adults. Ramadan fasting was found to be associated with beneficial effect on the lipids profile, fasting blood glucose, and body weight among healthy subjects [3].

The literature offers limited data about the safety of fasting Ramadan in patients with different renal diseases. Some studies have examined the effect of Ramadan fasting on physiological and biomedical markers in patients with kidney diseases [4]. While some studies advice against fasting in this special population, as patients with CKD and patients who are on HD carry a higher risk for dehydration during long fasting hours and, on the other hand, are at risk for fluid overload due to increased fluid intake when breaking fast after sunset, others dont [4].

Ramadan fasting was not associated with significant adverse effects in kidney transplant patients after 1 year of kidney transplantation or urinary risk factors for calculus formation. So, research findings on the safety of Ramadan fasting by patients with CKD on maintenance hemodialysis are mixed and controversial [3].

The aim of the current observational study was to monitor tolerability of Ramadan fasting in hemodialysis population and its effect on dry weight and general wellbeing of the participating patients.

## Patients and methods

### Study setting

A prospective cohort study conducted between April and May 2021 in Ain Shams University Hospital Dialysis Unit, Cairo, Egypt.

### Patients and study design

This study was held with the participation of 199 ESKD patients maintained on regular HD, 3 sessions per week, 4 h each using bicarbonate dialysate, with high flux dialyzers of surface area of 1.8 m^2^ or more and heparin as anticoagulation.

Ramadan occurred on April 12 to May 13; it was springtime in Egypt with an average temperature of 33°C, fasting hours were almost 15 h, and this was during the COVID-19 pandemic.

A hundred and ninety-nine prevalent hemodialysis patients participated in the study, aged 18 years or older. Patients were divided into 3 different groups based on their fasting state: group A, complete fasting (CF), comprising patients who decided to fast the whole month of Ramadan; group B, partial fasting (PF), comprised patients who planned to fast in the non-dialysis day; and group C, comprised patients who did not fast Ramadan (non-fasting) (NF). There was no influence from our side on patients’ intentions to fast or break-fast neither there was any modifications in patients’ usual dietary habits for the holy month.

Patients were asked to report any untoward side effects during their fasting hours, and they were closely monitored during their dialysis sessions for any complications. They were also instructed to stick to their treatment regimens during their non-fasting hours.

Basic demographic data, duration and access of hemodialysis, associated comorbid conditions, and viral state, in addition to patients’ residual renal function data and monthly laboratory tests (hemoglobin, calcium, phosphorus, albumin, iron status, and PTH level) were collected from the patients and their medical records. Baseline dry weight of all participating patients was assessed before the start of the month of Ramadan and monitored throughout till 1 week after the end of the holy month.

### Data management and analysis

The collected data was revised, coded, tabulated, and introduced to a PC using Statistical package for Social Science (SPSS 25). Data was presented and suitable analysis was done according to the type of data obtained for each parameter. Descriptive statistics included mean, standard deviation (± SD), and range for parametric numerical data, while it was median and interquartile range (IQR) for non-parametric numerical data and frequency and percentage of non-numerical data.

Analytical statistics included Student *T* test to assess the statistical significance of the difference between two study group means, mixed-design ANOVA test to assess the statistical significance of the difference between more than two study group means, post hoc test for comparisons of all possible pairs of group means, and paired *t*-test to assess the statistical significance of the difference between two means measured twice for the same study group. *P*-value: level of significance, *P* > 0.05: nonsignificant (NS), and *P* < 0.05: significant (S).

### Ethical considerations

All patients gave an informed consent to be enrolled in the study; also, the consent for publication was taken from all participants. The study follows the ethical considerations by the Research Ethical Committee of Faculty of Medicine, Ain Shams University, and was following the Helsinki Declaration as revised in 2000 with reference number FWA 000017585 (FAMSU R 95/2022)

## Results

This prospective study was conducted at the hemodialysis unit, Ain Shams University Hospitals, Cairo, Egypt. One hundred ninety-nine HD patients were included in the study (97 males, mean age 45 ± 15 SD) during the holy month of Ramadan from April 12 to May 13, 2021, which happened to occur during the COVID-19 pandemic.

Demographic data and clinical characteristics of the study population are shown in Table 1. Twenty-eight patients (14%) fasted the whole month, 88 (44%) partially fasted (they fasted in their non-dialysis days), with total of 116 fasting patients, while 83 (42%) did not fast. Among recorded comorbidities, HTN came in 1st rank (83 patients, 42.7%). Basic pre-fasting laboratory data of the study population are shown in Table 2.

**Table 1:** Basic demographic data and clinical characteristics of study population

**Characteristic**	**Mean (SD)/median (IQR)**
**Age**	45.78 (15.97)
**Duration of HD (years)**	6 (3–12)
**Dry weight** (pre-fasting)	67.77 ± 21.52
**Characteristic**	***N*** **(%)**
**Gender**	
Male	97 (48.7%)
Female	102 (51.3%)
**Vascular access**	
AVF	181 (91%)
Permanent catheter	16 (8%)
Temporary catheter	2 (1%)
**Type of fasting**	
No	83 (41.7%)
Complete	28 (14.1%)
Partial	88 (44.2%)
**Residual renal function (RRF)**	
Negative	173 (87.4%)
Positive	25 (12.6%)
**Virology (HCV)**	
Positive	55 (27.6%)
Negative	144 (72.4%)
**Comorbidities**	
HTN	83 (41.7%)
DM	23 (11.6%)
IHD	7 (3.5%)
Amyloidosis	2 9 (1.0%)
Heart failure	6 (3.0%)
AF	2 (1.0%)
Bronchial asthma	1 (0.5%)

*AVF* arteriovenous fistula, *HTN* hypertension, *DM* diabetes mellitus, *IHD* ischemic heart disease, *AF* atrial fibrillation

**Table 2:** Basic pre-fasting lab investigations for study population

	Mean (± SD)/median (IQR)
Hgb	10.14 ± 1.55
Ca	8.55 ± 0.96
Po4	4.39 ± 1.66
Alb	3.88 ± 0.40
iron	47.1 (34.5–70.6)
PTH	425 (191–706)
Ferritin	812.6 (279.2–1372)
TIBC	230.99 ± 71.82
T-SAT	21 (15–33)

*Hgb* hemoglobin, *Ca* calcium, *Po4* phosphorous, *Alb* albumin, *PTH* parathyroid hormone, *TIBC* total iron binding capacity, *T-SAT* transferrin saturation

Regarding fasting-related complications during the study period, only 4 patients (3.4%) developed complications as follows: 3 patients (2 CF and 1 PF) reported intradialytic hypotension (IDH), and one patient (PF) reported muscle cramps during their dialysis sessions. On the other hand, 3 patients experienced improvement of IDH; also, one patient reported improvement of his dyspepsia symptoms. The remaining 112 (96.6%) patients did not report any complications or events during the study period.

**Fig. 1:**
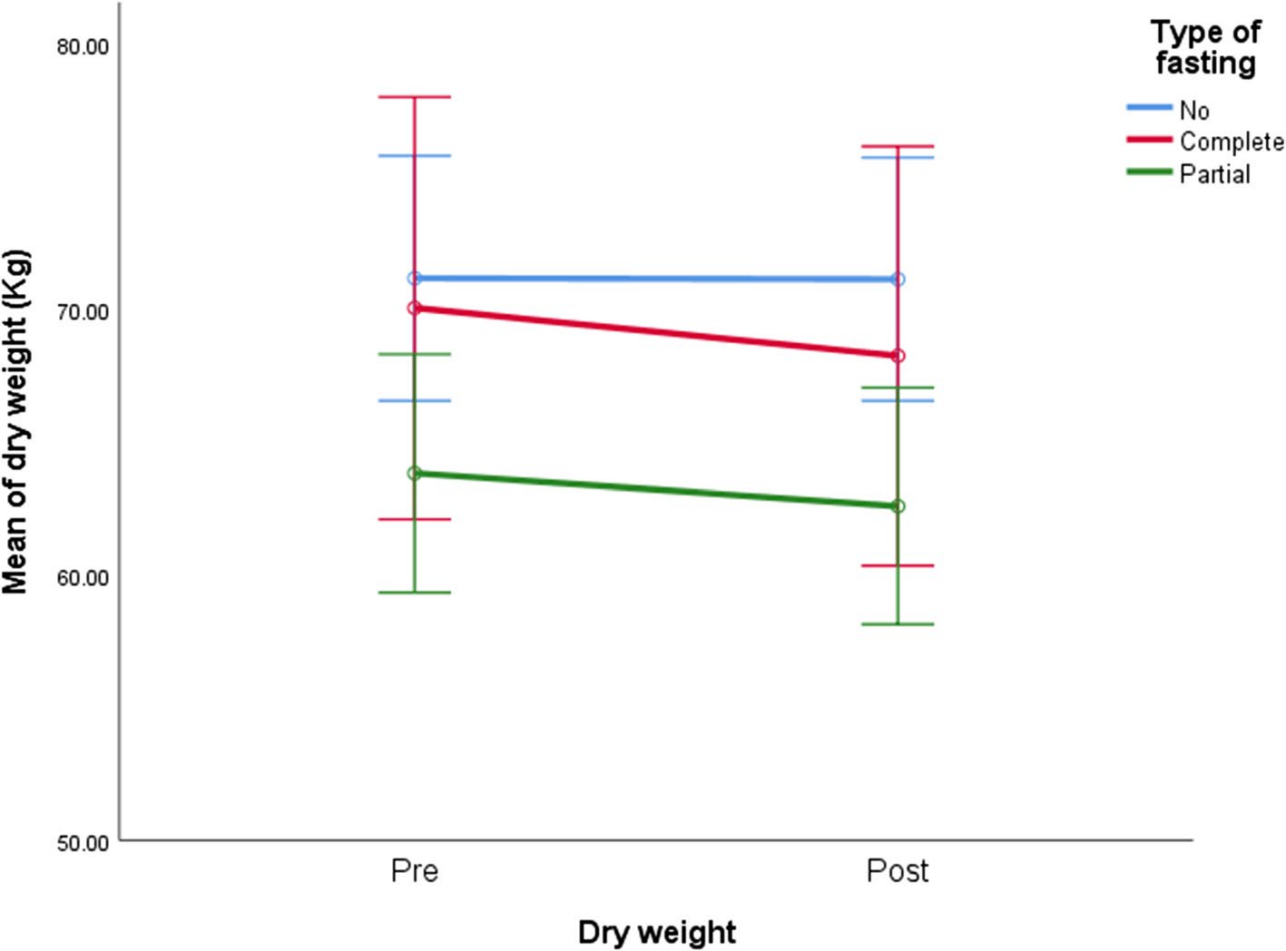
Weight changes in different fasting groups of study population pre- and post-fasting Ramadan

Weight changes pre- and post-fasting in the study groups are demonstrated in Table 3 and Fig. 1. A highly significant reduction in dry weight in the complete and partial fasting groups (*P* < 0.001 for both) was noted, unlike the non-fasting group, where no significant weight changes were noted (*P* = 0.75). However, no significant difference was noted in weight changes between complete and partial fasting groups at the end of the study (*P* = 0.22).

**Table 3:** Pre and post-fasting weight changes reported in the study groups

Dry weight (kg)	Type of fasting		
	Non	Complete	Partial
Pre (mean ± SE)	71.17 ± 2.34	70.05 ± 4.03	63.82 ± 2.27
Post (mean ± SE)	71.14 ± 2.32	68.25 ± 4	62.6 ± 2.26
Pairwise comparisons			
Mean diff. ± SE	− 0.04 ± 0.12	− 1.8 ± 0.2	− 1.23 ± 0.11
^*^P value	0.759	<0.001	<0.001

^*^Paired *t*-test

## Discussion

Our prospective study showed that fasting Ramadan in HD patients may be well tolerated by most of patients with no significant adverse effects, in addition to favorable effect on dry weight.

Limited data are available about the safety of fasting in Ramadan in patients with different renal diseases. Although some studies advice against fasting in this special population as patients with different degrees of renal disease, as they carry a higher risk for either dehydration during fasting hours or fluid overload due to increased fluid intake when breaking fast, some patients choose to overlook these recommendations, and still, they do fast Ramadan for its spiritual spirits and religious reasons [4].

Previously held studies showed different percentages of their patients allocated among either the complete or partial fasting state compared to the results of the current study. One study by Megahed and colleagues stated that among the 2055 included Muslim HD population, 965 (46.96%) succeeded to fast, of those who fasted, 39.5% admitted that they could fast the whole month [2]. In a previous report, 282 Pakistani HD patients were assessed by Imtiaz et al., and the frequency of fasting in Ramadan was 13.5% [5]. In another study conducted in Saudi Arabia, 64.1% out of the included 635 HD patients fasted [6]. The variation in the results of these studies and the current one could generally be ascribed to different personal, social, and environmental influences.

Body weight changes during Ramadan has always been a debate, between weight loss, gain, or even no change, particularly in special patients’ groups. In the current study, we noticed a significant reduction in the body weight among the fasting groups of patients after the holy month of Ramadan: 1.8 kg in the CF group (*P*-value: ˂ 0.001), 1.23 kg in the PF group (*P*-value: ˂ 0.001), unlike patients who did not fast, where they did not show a significant change in their weight during the holy month (*P*-value: 0.75).

This result is further supported by the results by Wan Md Adnan and colleagues, where they stated a significant decrease in the weight of patients who voluntary fasted during Ramadan [7]. Moreover, Bernish et al. confirmed the same finding stating that the main positive clinical finding, in their study, was the tendency towards weight reduction (1.4) kg and the decrease in systolic and diastolic blood pressure [4].

Conversely, other studies that investigated the same matter showed either no change in the patients’ body weight as noted by Megahed et al. and Elshamsi et al. [2, 8] or even reported weight gain during the holy month for the fasting dialysis patients as in the study by Khazneh et al. who reported a slightly higher mean IDWG by 0.6 kg and 0.4 kg in the complete and partial fasting groups compared to the non-fasting, respectively [9].

Those mixed findings might be attributed to the different cultural and festive traditions in different nations during the holy months in which, of course, food represents a big part.

With regard to safety of Ramadan fasting in hemodialysis population, in the current study, fasting Ramadan whether complete or partial was safe and tolerated by 96.6% of the patients (112/116). Reported untoward events occurred in 3.4% of patients, mostly hypotension along with muscle cramps. Wan Md Adnan et al. results agreed with ours in their previously mentioned study, stating that all patients who participated in this study tolerated Ramadan fasting quite well [7].

However, conflicting with this result was the results by Rashed, who noted that patients on long-term HD who fasted during Ramadan may experience an increase in body weight and fluid overload between dialysis sessions due to their tendency to increase food consumption at nights of Ramadan [10].

Moreover, in the results of Khazneh et al., in their previously mentioned study, although they stated that Ramadan fasting was not associated with significant complications, they highlighted that these patients are at higher risk of hyperkalemia and volume overload and mandated that hemodialysis patients intending to fast should be aware of the consequences of hyperkalemia and volume overload and should strictly adhere to their allowance of fluid intake and potassium rich diets [9].

## Conclusion

We may conclude from our study that fasting Ramadan in hemodialysis patients whether complete or partial fasting may be tolerated by most of patients without significant complications. Moreover, significant reduction in dry weight was noted in the fasting group which may be an added benefit. Of note, once fasting decision is made by these patients, it should be thoroughly discussed with them, going over the potential risks of fluid overload and hyperkalemia and highlighting the importance of adhering to their dietary and fluid allowances. Moreover, treating physicians should monitor these patients during their sessions for any untoward adverse effects, if any.

## Data Availability

All data and materials used in the study will be available whenever requested.
